# Supplementary material for: Video triage of children with respiratory symptoms at a medical helpline is safe and feasible–a prospective quality improvement study
Source: PLoS One. 2023 Apr 19;18(4):e0284557. doi: 10.1371/journal.pone.0284557 (PMC10115256; doi:10.1371/journal.pone.0284557)

Appendix 1. Flowchart of project workflow during video- and telephone triage calls, depicting the call-handlers' workflow.

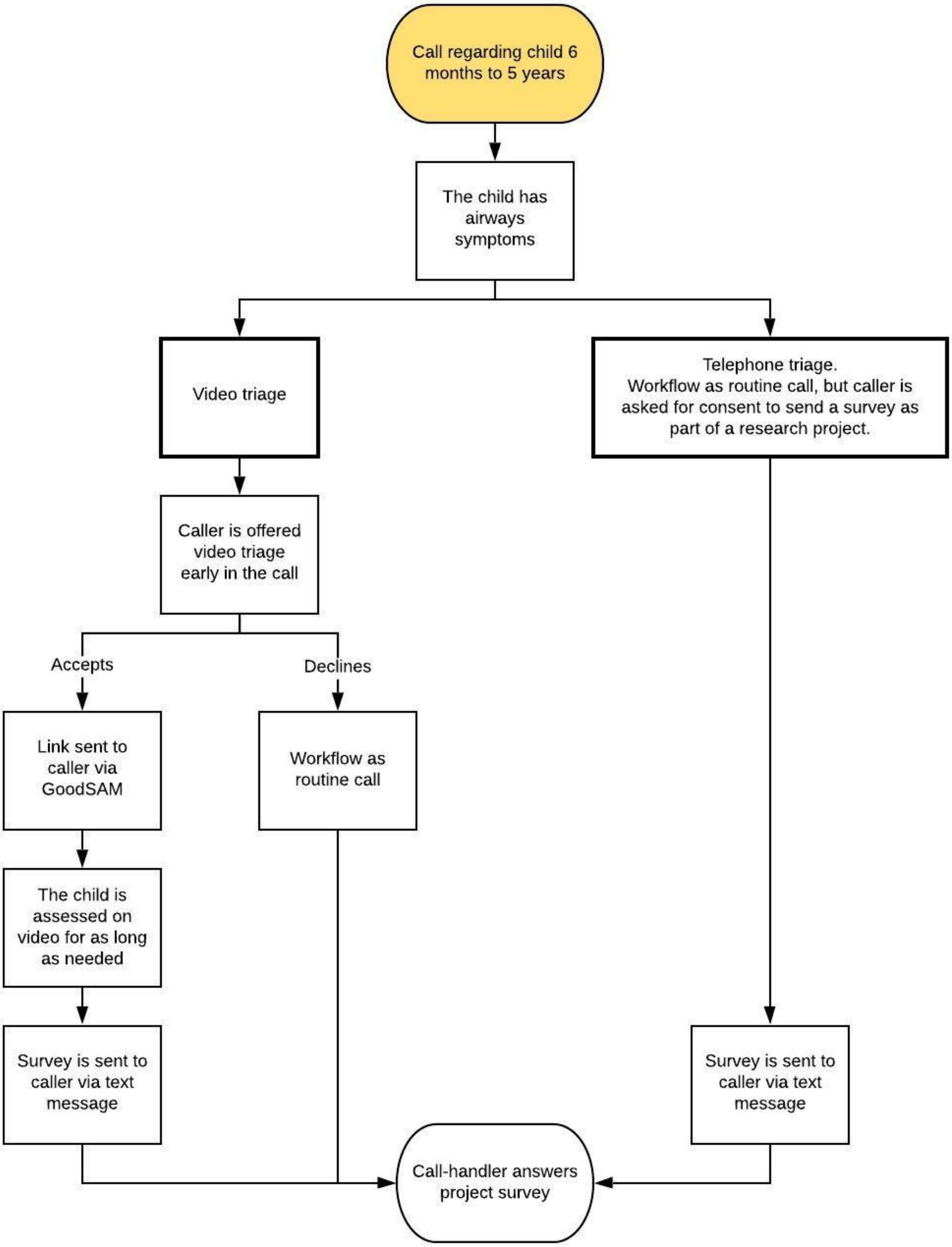

Supplement: S1 Appendix — (PDF) [file pone.0284557.s001.pdf]
