# Supplementary material for: Video triage of children with respiratory symptoms at a medical helpline is safe and feasible–a prospective quality improvement study
Source: PLoS One. 2023 Apr 19;18(4):e0284557. doi: 10.1371/journal.pone.0284557 (PMC10115256; doi:10.1371/journal.pone.0284557)
Supplement: S2 Appendix — Patient outcome with and without pilot group included. *Chi square test. (PDF) [file pone.0284557.s002.pdf]

Appendix 2. Sensitivity analysis. Patient outcome with and without pilot group included.

	Video triage	Telephone triage	p-value*
**Pilot group excluded**	n=250	n=204	
**Response,** *percentage (number)*<br>Staying home<br>Hospital	 63% (157/250)<br>37% (93/250)	 57% (117/204)<br>43% (87/204)	 0.24
**Patients assessed at hospital within 48 hours after call,** *percentage (number)*	47% (118/250)	51% (105/204)	0.37
**Outcome at hospital,** *percentage (number)*<br>Sent home after assessment without paraclinical testing/treatment/prescription/hospitalization<br>Received treatment and/or paraclinical testing and/or hospitalized	 41% (48/118)<br>59% (70/118)	 46% (48/105)<br>54% (57/105)	 0.45
**Pilot group included**	n=336	n=281	
**Response,** *percentage (number)*<br>Staying home<br>Hospital	 63% (213/336)<br>37% (123/336)	 58% (163/281)<br>42% (118/281)	 0.17
**Patients assessed at hospital within 48 hours after call,** *percentage (number)*	46% (155/336)	51% (143/281)	0.24
**Outcome at hospital,** *percentage (number)*<br>Sent home after assessment without paraclinical testing/treatment/prescription/hospitalization<br>Received treatment and/or paraclinical testing and/or hospitalized	 41% (64/155)<br>59% (91/155)	 46% (66/143)<br>54% (77/143)	 0.40

*Chi square test
