# Supplementary material for: Video triage of children with respiratory symptoms at a medical helpline is safe and feasible–a prospective quality improvement study
Source: PLoS One. 2023 Apr 19;18(4):e0284557. doi: 10.1371/journal.pone.0284557 (PMC10115256; doi:10.1371/journal.pone.0284557)
Supplement: Attachment — Submitted filename: response to reviewers.docx [file pone.0284557.s003.docx]

Dear Richard Bruce Mink

On behalf of all the authors, I thank you for your interest in our manuscript ”Video triage of children with respiratory symptoms at a medical helpline is safe and feasible – a prospective quality improvement study” (PONE-D-21-06235).

We are today submitting a revised version, changed according to your points raised. Thus, we have moved all data regarding user satisfaction to the accompanying paper (“We can’t do without it”: parent and call-handler perceptions on video triage of children at a medical helpline, PONE-D-21-08760), and this present paper now solely focuses on patient outcome. We have also changed wordings such as *decreased* and *increased* when the statistical tests do not support it. The additional requirements, such as style requirements and consent forms for clinical pictures have also been fulfilled. We would like to underline the fact that, as mentioned in the manuscript, sharing of sensitive patient data originating from quality improvement data is prohibited in the Danish legislation, but a minimal data set has been prepared and can be requested by addressing the contact person mentioned in the manuscript.

We sincerely hope that this revised manuscript is accepted for publication, as we believe that it makes an important contribution to the knowledge of using video streaming in health services, a tool getting steadily increased focus worldwide.

Kind regards,

*Dr. Caroline Gren*
