# Supplementary material for: Video triage of children with respiratory symptoms at a medical helpline is safe and feasible–a prospective quality improvement study
Source: PLoS One. 2023 Apr 19;18(4):e0284557. doi: 10.1371/journal.pone.0284557 (PMC10115256; doi:10.1371/journal.pone.0284557)
Supplement: Attachment — Submitted filename: response to reviwers 180622.docx [file pone.0284557.s004.docx]

| **Reviewer’s comments** | **Authors’ responses** |
| --- | --- |
| Reviewer #1: Thank you for the opportunity to review this manuscript. I was not a reviewer for the initial submission but have read both versions. This is a great study concept and design. Congratulations on this very important work. See specific minor edits below. Line number references are from the revised document with Track Changes. | Thank you for your time and well thought-through comments. We have now tried to revise the manuscript according to your comments, and hope that you find the new version acceptable. |
| One primary concern I have is with the use of the word “feasible” – in that nearly 10%, or 70 of the 734 calls included, either declined video triage or were unsuccessful in video streaming (data is from Figure 1). My concern is that those who declined may have done so due to lack of smartphone (line 132) or other device, or reliable internet to achieve this. Either way, it appears that video is not feasible for ~10% of the population sampled. This should be commented on in the discussion. | We have added definitions of acceptability and feasibility in the Outcomes paragraph in the Methods section. Hopefully this helps. We have also altered the calculations of feasibility and acceptability rates, due to this very reasonable comment of yours, and both are now at 9%. We believe, that 9% is a good number considering this was a new tool, and also because of the possibly stressful situation of sick children for both call-handlers and parents. Lines 305-310 and 349-352 discusses this area.  We did not instruct the call-handlers to ask parents about why they did not wish to participate. However, the prevalence of smart-phones in Denmark is extremely high (>90%, <https://www.dst.dk/en/Statistik/temaer/SDG/globale-verdensmaal/05-ligestilling-mellem-koennene/delmaal-b/indikator-1>), especially so in this age group, as is the prevalence of Internet access in private homes, so this should not be an issue. Furthermore, although we did not specifically investigate it, we did not receive any questionnaire responses from parents stating that were not able to participate due to bad Internet access or lacking smartphone. |
| Additionally, for the ones triaged to video, can you please comment on how many were started as video and then could not be successfully completed? This is partially addressed in Figure 1, but could be improved. I do recommend that you write about this relevant detail from this figure in the manuscript narrative, and not only refer to the Figure. | Yes, we agree that this is important, and it is mentioned in lines 215-216 in manuscript with tracked changes, and we have also added this in the discussion at lines 305-310. |
| Please provide brief details about the intervention beyond “the technology is described in detail elsewhere” – it would serve the readers to have a brief summary, even if one sentence, for clarity in this manuscript | Yes, you are completely right, and we have therefore described the technology in greater detail in the paragraph at lines 105-108. |
| Patient outcome (line 242): in this section, it is not clear to the reader whether within 8 hours the patients assessed in the hospital were because of the recommendation of the triaging call/video. Please be more clear about proportion of patients who were triaged to each outcome versus those who were triaged to one outcome but then presented to the hospital anyway - because of clinical condition or other; this needs to be fleshed out in the discussion as well, and would be beneficial in the paragraph starting at line 333 | A short paragraph has been added at the start of this section; lines 224-227, where we state these results comprise all patients, irrespective of initial triage outcome. Lines 244-247 also discusses this topic, where we present that 57 of patients seen at hospital had initially been advised to stay at home (57/298=19%).We also added the lines 298 and 299 in the discussion. |
| The results presented do not support that added assertion that video is “acceptable” (conclusion, line 432), given that parent and provider perspectives are removed from this paper. | Yes, we agree that the users’ experiences and perspectives are described in the qualitative paper related to this study. However, we have now added definitions of acceptability and feasibility in the Outcomes paragraph in the methods section, and also the updated rates, now both at 9%. We hope this is acceptable. |
| Otherwise, I do recommend an additional review for grammar and syntax. For instance, make sure that sentences that start with numbers have them spelled out (e.g. line 235 “There 63% of … “ should read “Sixty-three percent of…” and delete the “There”). Another example is the parenthetical reference to a different paper that you have submitted (line 85 and line 163, “reference to accompanying paper”). There are other grammatical errors throughout the manuscript, please review and correct. | Thank you for this comment. and the manuscript has now undergone revisions by a professional proofreader. The mentioned reference har now been changed, as the qualitative paper has been published in the meantime. |
