# Supplementary material for: Video triage of children with respiratory symptoms at a medical helpline is safe and feasible–a prospective quality improvement study
Source: PLoS One. 2023 Apr 19;18(4):e0284557. doi: 10.1371/journal.pone.0284557 (PMC10115256; doi:10.1371/journal.pone.0284557)
Supplement: Attachment — Submitted filename: response to reviewers 191222.docx [file pone.0284557.s005.docx]

| Comment by reviewer | Response from authors |
| --- | --- |
| Thank you for inviting me to review this manuscript entitled on: Video triage of children with respiratory symptoms at a medical helpline is safe and feasible – a prospective quality improvement study. The study aimed at to study the safety and feasibility of introducing video triage of young children with respiratory symptoms at a medical call center, as well as the impact on patient outcome. The authors have raised very important issue. After the authors address the following concerns, it will be suitable for publication to Plose one journal | We thank you for your time and effort in reviewing our manuscript, and we hope that you will find our comments and corrections satisfying. We too find this an important issue that deserves attention. |
| The abstract is somehow complex to understand. It would be better if authors make it clear. It lacks some points which must be included in the method section of the abstract. It doesn’t sate clearly about the data collection tools, study period, final sample size, study design and model of data analysis | We have now added the wanted information (data collection tools, study period, sample size and data analysis) to the abstract.  Final sample size: 617; study design: prospective quality improvement study with patients enrolled to video or standard telephone triage (1:1); data analysis: “Logistic regression was used to test the effect on outcomes.” (this is the main method used); and finally, study period (February 2019-March 2020) has been added to abstract and results section, thank you for pointing this out. |
| Why children’s less than six months are excluded from the study? | Yes, this is a relevant question, thank you. We chose 6 months as a cut-off due to infants relatively more often having severe illness, and we therefore did not want to take any chances in this early phase, before we were sure that video triage was safe. Also, infants are inherently harder to assess than older children, and are generally recommended to be assessed face-to-face. We have added a section about this at lines 123-126. |
| Explain how the limitation of this study affect your result. | Yes, this is a very relevant discussion. The most important limitation, that the study was not conducted as a randomized controlled study, due to the call-handlers not having time to inform the patients sufficiently for a written informed consent, and difficulties of setting the computers up to do the randomization, conveys the possibility that the two groups (video and telephone triage groups) are not completely alike. But as age, gender, symptoms and hospital outcome are similar, we reckon that the groups are comparable. But of course, a randomized controlled trial would have been preferable.  Another important limitation is that we did not reach the calculated sample size, due to the Corona pandemic. Had we reached the sample size calculated beforehand, we believe that we would have reached a significant result concerning how many patients that were assessed at hospital after the call. Lastly, we cannot know how video triage works in patients in other ages or with other symptoms, this must be tested in other studies, and that would be interesting to investigate. However, we tested video triage in the most frequently occurring pediatric population at this medical helpline, i.e. young children with symptoms from the airways, so the results should thus be representative for a large part of pediatric calls.  This discussion can be found in the limitations-section as well. |
| How the authors did model testing? if no justify, if yes state clearly | We tested the model during the first two weeks of the study, the pilot phase, where we evaluated how the nurses experienced the set-up and how the technical solution worked. Afterwards we made some smaller adjustments.  Also, before this study was launched, a study at the Danish emergency helpline was using the same technical set-up (GoodSAM instant-on-scene) and we therefore could use some the experiences gained from that study. |
| How is the validity of the data collection tool? was it validated? if yes reference, if no justify | We are not completely certain what is understood by “data collection tool” in this context. However, the surveys answered by the call-handlers were not validated more than by oral feedback from the call-handlers during the pilot period regarding for example clarity of wording.  Concerning the internal electronic patient records at MH1813, they are used every day and maintained by a data management department, and data concerning all the participating children was found and was complete.  Data collection from the hospitals’ electronic charts were done manually by the first author, and all children were identified, and all wanted data was able to be obtained. Generally, Danish registers are renowned to be complete and reliable. |
